# Supplementary material for: The cell cycle inhibitor RB is diluted in G1 and contributes to controlling cell size in the mouse liver
Source: Front Cell Dev Biol. 2022 Aug 25;10:965595. doi: 10.3389/fcell.2022.965595 (PMC9452963; doi:10.3389/fcell.2022.965595)
Supplement: Supplementary file 1 [file Image1.pdf]

Supplementary Material

Supplementary figure 1

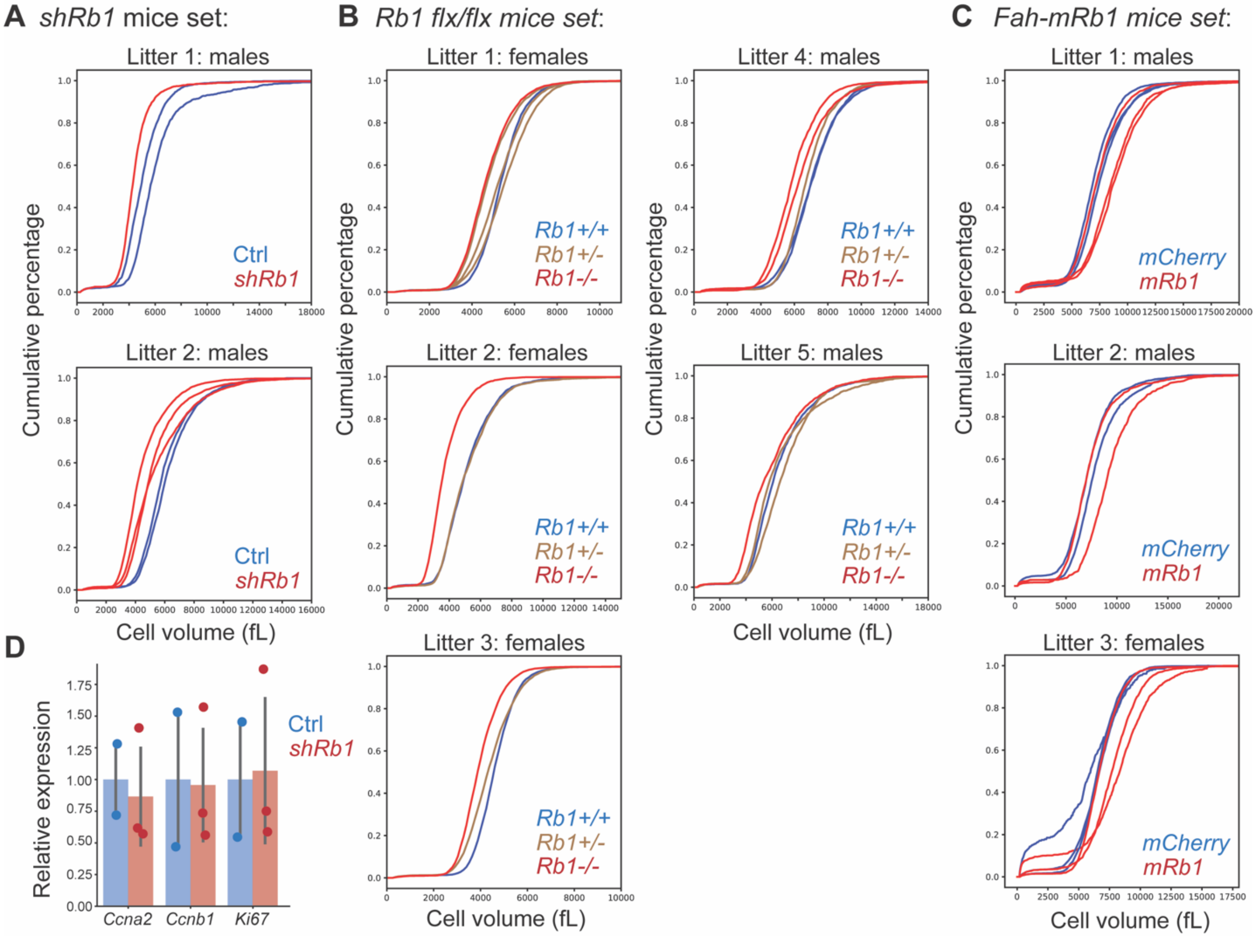

Figure S1: Cell size distributions of hepatocytes in different mice experiments.
The cumulative size distribution of the tetraploid hepatocytes measured by Coulter counter. Each panel shows the mice from the same litter. (A) is from the *shRb1* mice experiment, (B) is from the liver specific knockout mice, and (C) is from the *Fah-/-* mice with transposon injection. D. qPCR measurement for cell cycle genes in the Litter 2 of *shRb1* mice set (as shown in A).

Supplementary figure 2

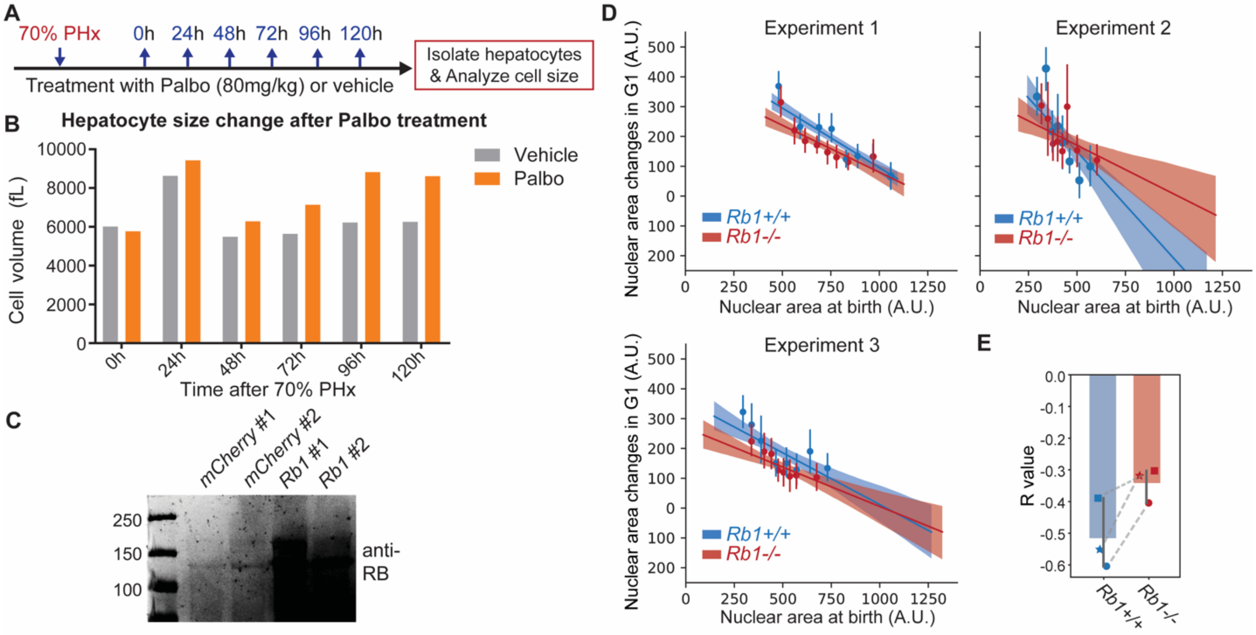

Figure S2: Cell size analysis following hepatectomy, and single cell time lapse imaging data for each individual experiment.

A. Schematic of the partial hepatectomy (PHx) experiment examining the effect of Palbociclib treatment. B. Coulter counter cell size measurements of hepatocytes isolated from mice at the indicated time points following surgery. On day 0, 1, 2, 3, 4, 5 after surgery, one mouse from each treatment was sacrificed to isolate primary hepatocytes. After isolation, cells were fixed and sorted based on their live/dead staining and their DNA staining. The tetraploid (the biggest population) cells were sorted and their sizes were measured using a Coulter counter. C. The same western blot as Figure 2D, but overexposed to show the RB band in mCherry mice. D The correlations between nuclear area at birth and the nuclear area changes during G1 in *Rb1*$^{+/+}$ and *Rb1*$^{-/-}$ hepatocytes from the three independent biological replicates. Each experiment used hepatocytes from different mice, and *Rb1*$^{+/+}$ and *Rb1*$^{-/-}$ hepatocytes for each replicate experiment were derived from the same mouse. The shaded area indicates the 95% confidence interval. Data were binned based on nuclear area at birth, and the mean and standard deviation of each bin is plotted. E. The R values of the linear correlations in (D) are plotted. Dashed lines link experiments performed using hepatocytes derived from the same mouse.
